# Supplementary material for: 4-Acetyl-Antroquinonol B Improves the Sensitization of Cetuximab on Both Kras Mutant and Wild Type Colorectal Cancer by Modulating the Expression of Ras/Raf/miR-193a-3p Signaling Axis
Source: Int J Mol Sci. 2021 Jul 14;22(14):7508. doi: 10.3390/ijms22147508 (PMC8307961; doi:10.3390/ijms22147508)
Supplement: Supplementary file 1 [file ijms-22-07508-s001.zip › ijms-1240086-supplementary.pdf]

SUPPLEMENTARY INFORMATION

Supplementary Table S1. Western blot antibodies sheet.

No.	Target	Dilution	Catalog	kDa
1	GAPDH	1:1000	GAPDH (0411K) Mouse mAb SC-27724	37
2	RAS	1:500	Ras (D2C1) Rabbit mAb #8955	21
3	KRAS	1:500	Proteintech Anti-KRAS Polyclonal, Catalog # 12063-1-AP	21
4	p-ERK	1:1000	Phospho-p44/42 MAPK (Erk1/2) (Thr202/Tyr204) Antibody #9101	42,44
5	p-MEK	1:1000	Phospho-MEK1/2 (Ser217/221) Antibody #9121	45
6	c-RAF	1:500	c-Raf Antibody #9422	65-75
7	p-c-RAF	1:500	Phospho-c-Raf (Ser259) Antibody #9421	74
8	Caspase 3	1:500	Anti-Caspase 3 antibody (GTX110543) GeneTex	32
9	Caspase 9	1:500	Caspase-9 Antibody (Human Specific) #9502	35,37,47
10	p-EGFR	1:500	Phospho-EGF Receptor (Tyr1173) (53A5) Rabbit mAb #4407	175
11	BRAF	1:500	Anti-BRAF antibody (ab85972)	84
12	PARP1	1:500	Anti-PARP1 antibody [EPR18461] (ab191217)	113

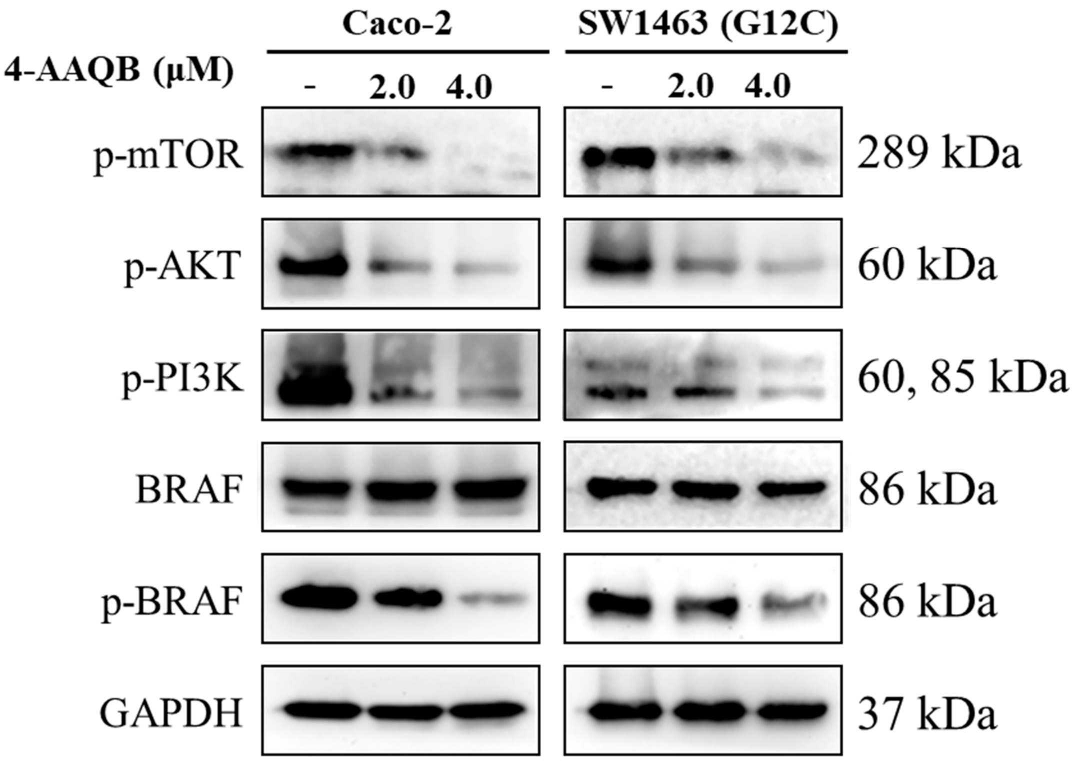

Supplementary Figure S1. Reduction in the oncogenic marker expression (p-mTOR, p-AKT, p-PI3K), and B-RAF/p-B-RAF was observed after the 4-AAQB treatment on both the CRC cells (SW1463 and Caco-2).

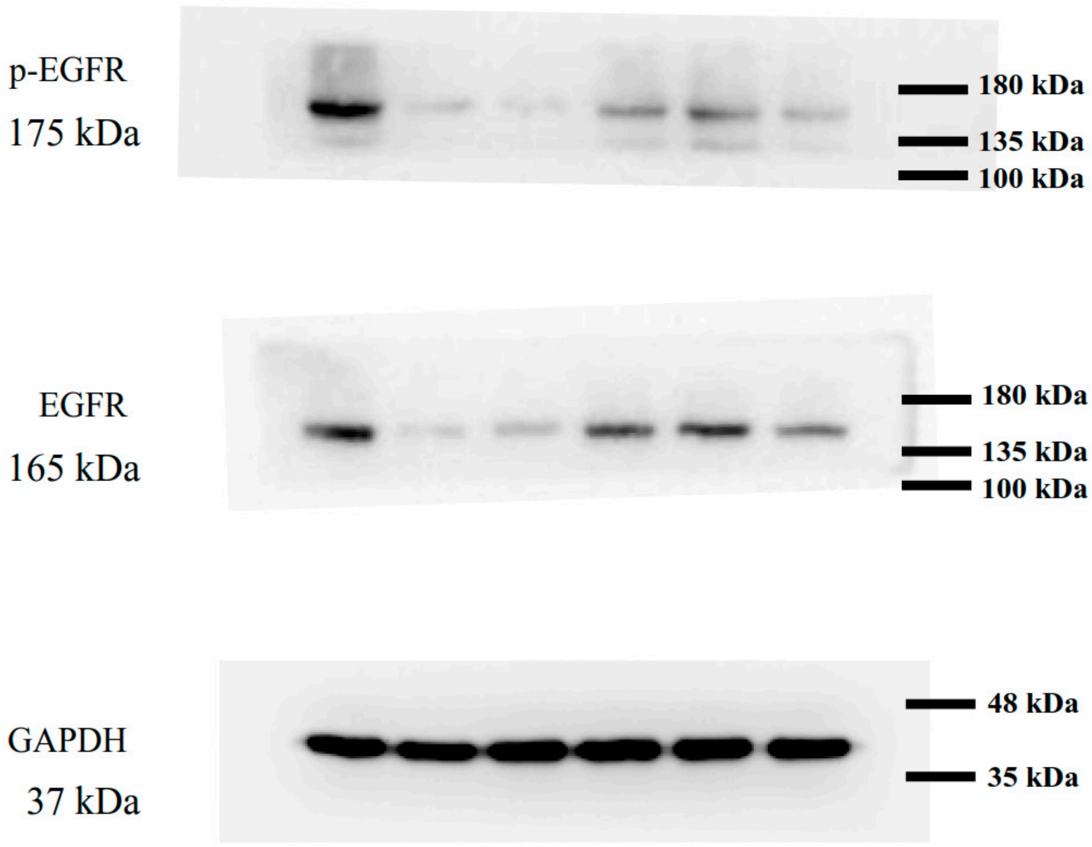

Supplementary Figure S2. Full-size blots of Figure 2A

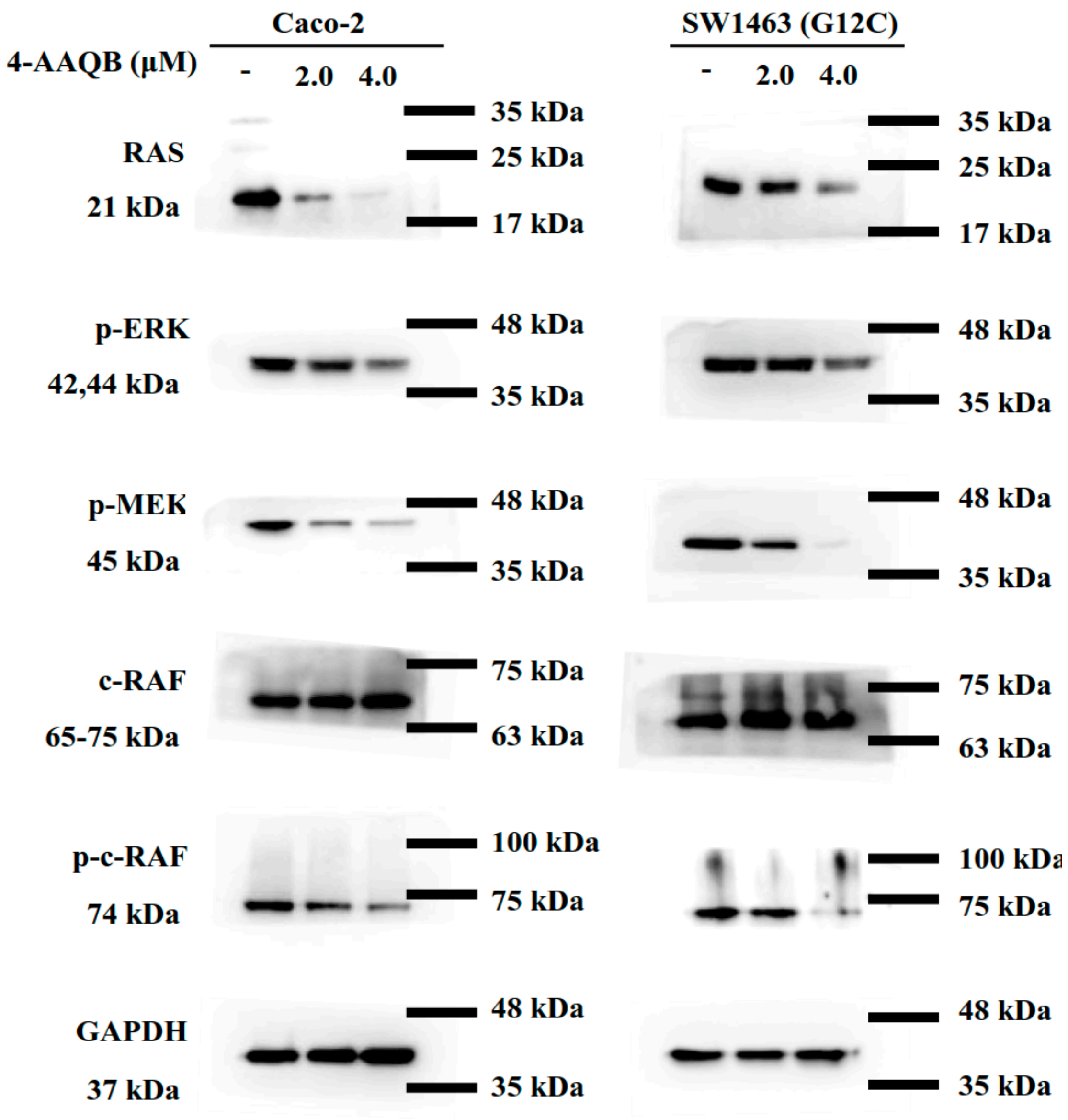

Supplementary Figure S3. Full-size blots of Figure 3H

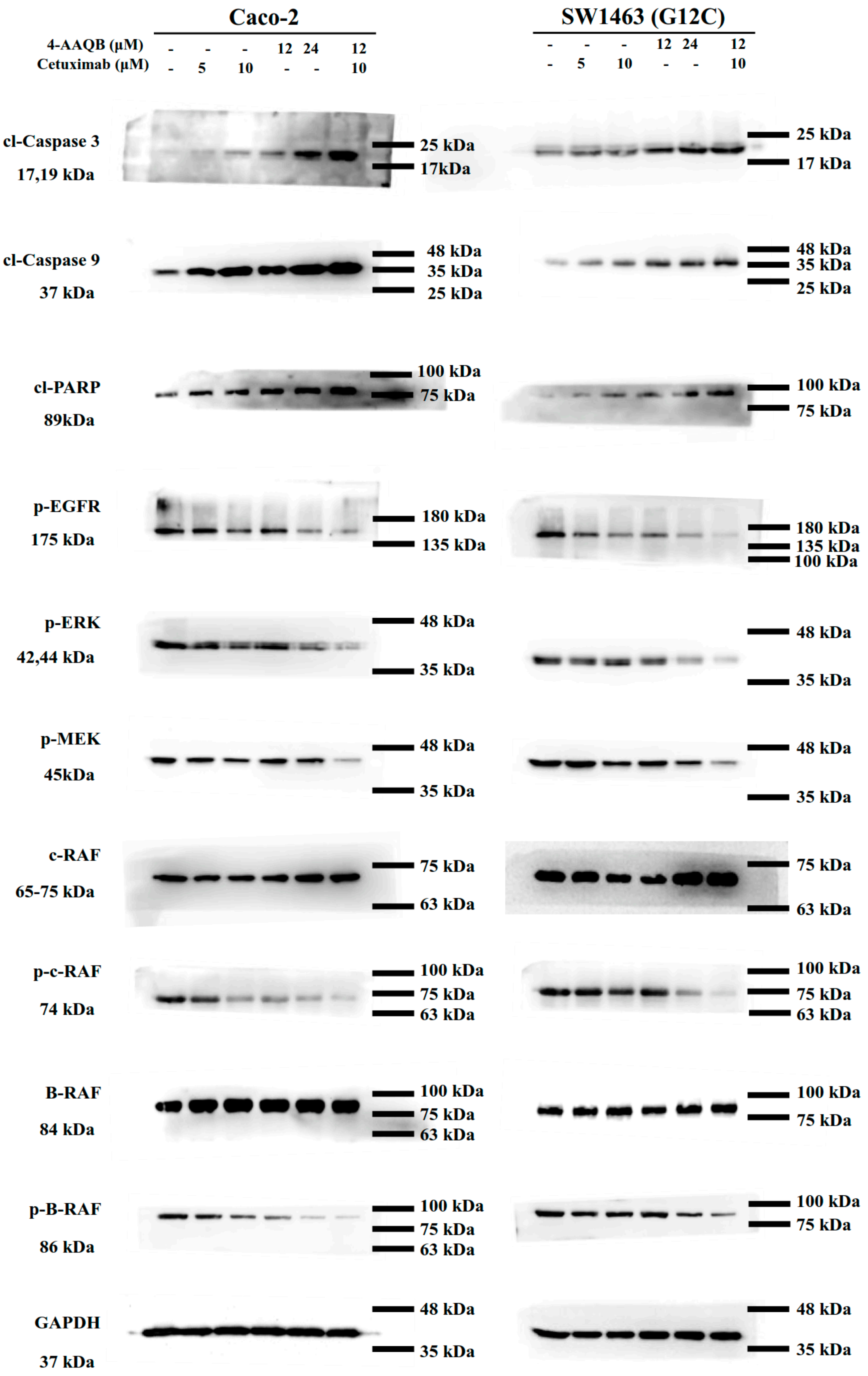

Supplementary Figure S4. Full-size blots of Figure 4F

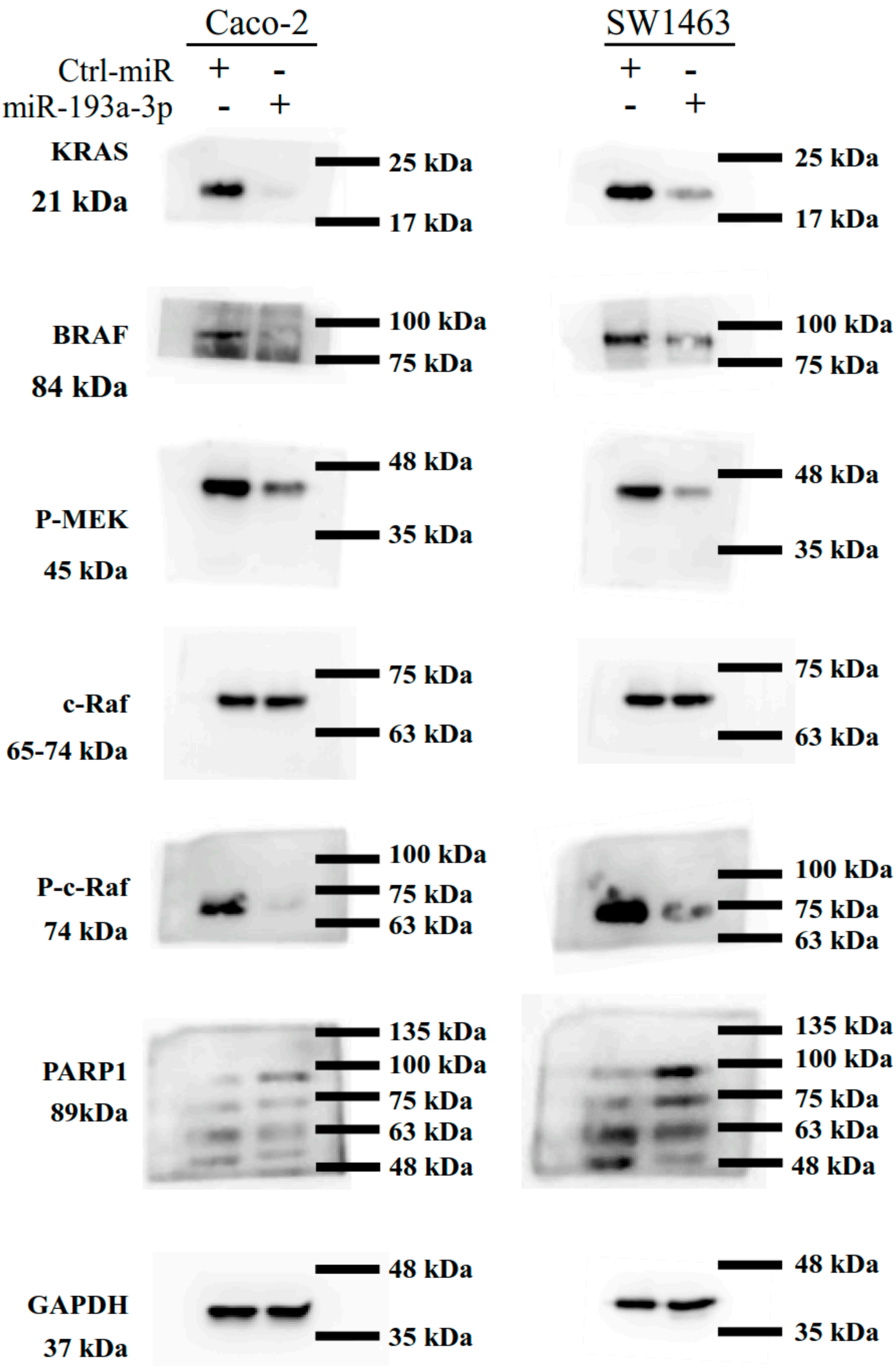

Supplementary Figure S5. Full-size blots of Figure 5H

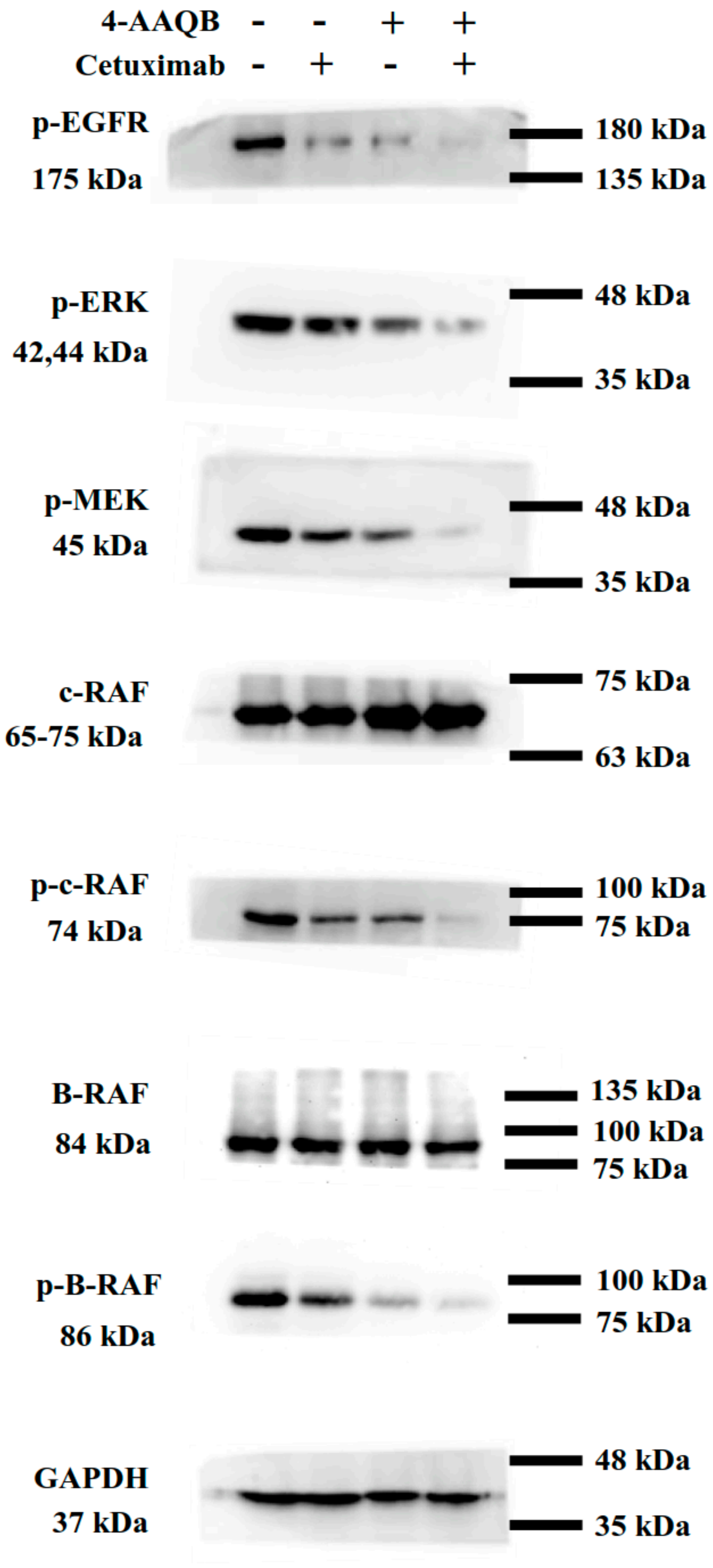

Supplementary Figure S6. Full-size blots of Figure 6C

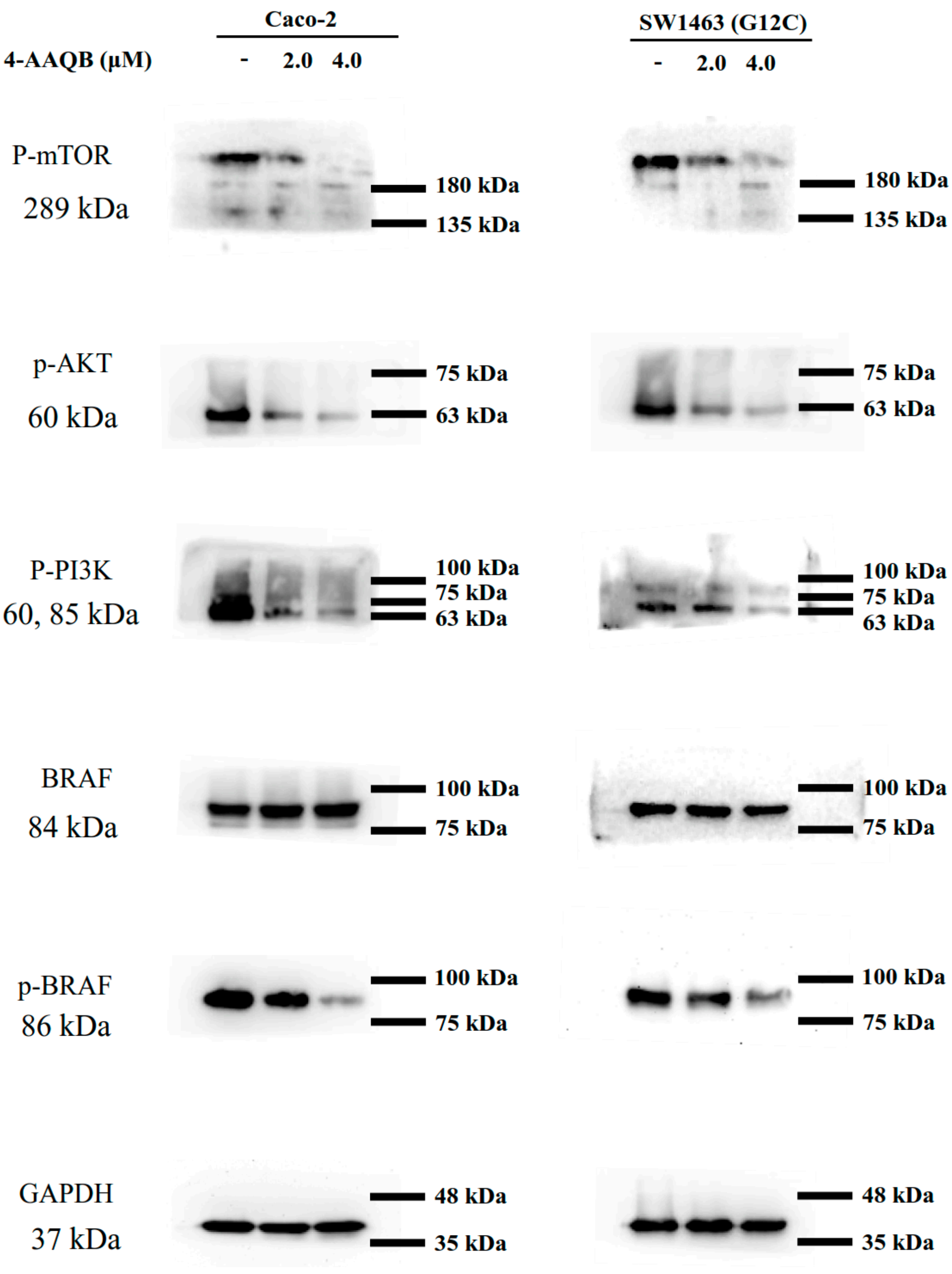

Supplementary Figure S7. Full-size blots of Supplementary Figure S1
